# Current landscape of cystic fibrosis gene therapy

**DOI:** 10.3389/fphar.2024.1476331

**Published:** 2024-10-08

**Authors:** Lindsey W. Plasschaert, Kelvin D. MacDonald, Jeffrey S. Moffit

**Affiliations:** ^1^ Plasschaert Consulting, Cambridge, MA, United States; ^2^ Carbon Biosciences, Waltham, MA, United States; ^3^ Department of Pediatrics, Oregon Health and Science University, Portland, OR, United States

**Keywords:** cystic fibrosis, CFTR, gene therapy, viral vector, airway, transduction

## Abstract

Cystic fibrosis is a life-threatening disease that is caused by mutations in *CFTR*, a gene which encodes an ion channel that supports proper function of several epithelial tissues, most critically the lung. Without CFTR, airway barrier mechanisms are impaired, allowing for chronic, recurrent infections that result in airway remodeling and deterioration of lung structure and function. Small molecule modulators can rescue existing, defective CFTR protein; however, they still leave a subset of people with CF with no current disease modifying treatments, aside from lung transplantation. Gene therapy directed to the lung is a promising strategy to modify CF disease in the organ most associated with morbidity and mortality. It is accomplished through delivery of a CFTR transgene with an airway permissive vector. Despite more than three decades of research in this area, a lung directed gene therapy has yet to be realized. There is hope that with improved delivery vectors, sufficient transduction of airway cells can achieve therapeutic levels of functional CFTR. In order to do this, preclinical programs need to meet a certain level of CFTR protein expression *in vitro* and *in vivo* through improved transduction, particularly in relevant airway cell types. Furthermore, clinical programs must be designed with sensitive methods to detect CFTR expression and function as well as methods to measure meaningful endpoints for lung structure, function and disease. Here, we discuss the current understanding of how much and where *CFTR* needs to be expressed, the most advanced vectors for *CFTR* delivery and clinical considerations for detecting CFTR protein and function in different patient subsets.

## 1 Introduction

Cystic fibrosis (CF) is the most common autosomal recessive disease and affects approximately 40,000 people in the United States and 80,000 people worldwide (Cromwell et al., 2023). It is caused by variants in the *Cystic Fibrosis Transmembrane Conductance Regulator (CFTR)* which encodes a chloride channel that is found on the membrane of many cell types in the body (Riordan, 1989; Saint-Criq and Gray, 2017). Lack of CFTR function at the apical surface of secretory epithelial tissues such as the lung and pancreas is the cause of morbidity, although many organs are affected including the reproductive system, intestine, liver, bone, and kidney. In the classic CF disease progression, pulmonary complications as a result of thick inspissated mucus, chronic bacterial colonization and ongoing lung destruction are the principal cause of death. The average life span of people with CF (PwCF) has drastically improved over the years to mid to late 40s (mid-50s if born after 2018) but has historically struggled to get beyond childhood without therapeutic intervention (Castellani and Assael, 2017; McBennett et al., 2022). A person with CF experiences an annual decline in lung function of 1%–2%, and without a lung transplantation or disease-modifying therapy, 80% will succumb to respiratory failure (Lyczak et al., 2002; Leung et al., 2020). Significant understanding of the genetic basis for CF and the resulting pathophysiology has led to the development of CFTR modulators, small molecules that correct and/or potentiate dysfunctional CFTR protein to clinically meaningful output with daily medication (Middleton et al., 2019). However, there is still great unmet need for PwCF that are unable to benefit from modulators due to their specific *CFTR* variant, intolerable side effects or lack of access (10%–20% of patients) (Hubert et al., 2017; Burgener and Moss, 2018). Therefore, a gene therapy for CF which introduces a functional *CFTR* gene into patients’ cells could treat >4,000 patients in the US unable to use modulators. Despite identification of the genetic cause more than three decades ago, *CFTR* gene therapy has yet to progress beyond clinical trials due to a lack of sustained efficacy and prolonged lung function improvements in patients. New insights into the expression and function of CFTR in relevant cell types, novel delivery methods for introducing *CFTR* into target airway cells and more sensitive measurements for lung function in the clinic, particularly in young children or those patients with milder disease most likely to benefit from early CFTR intervention, are promising steps toward a clinically approved CF gene therapy.

## 2 Genetic basis for cystic fibrosis disease and continuing unmet need

**FIGURE 1:**
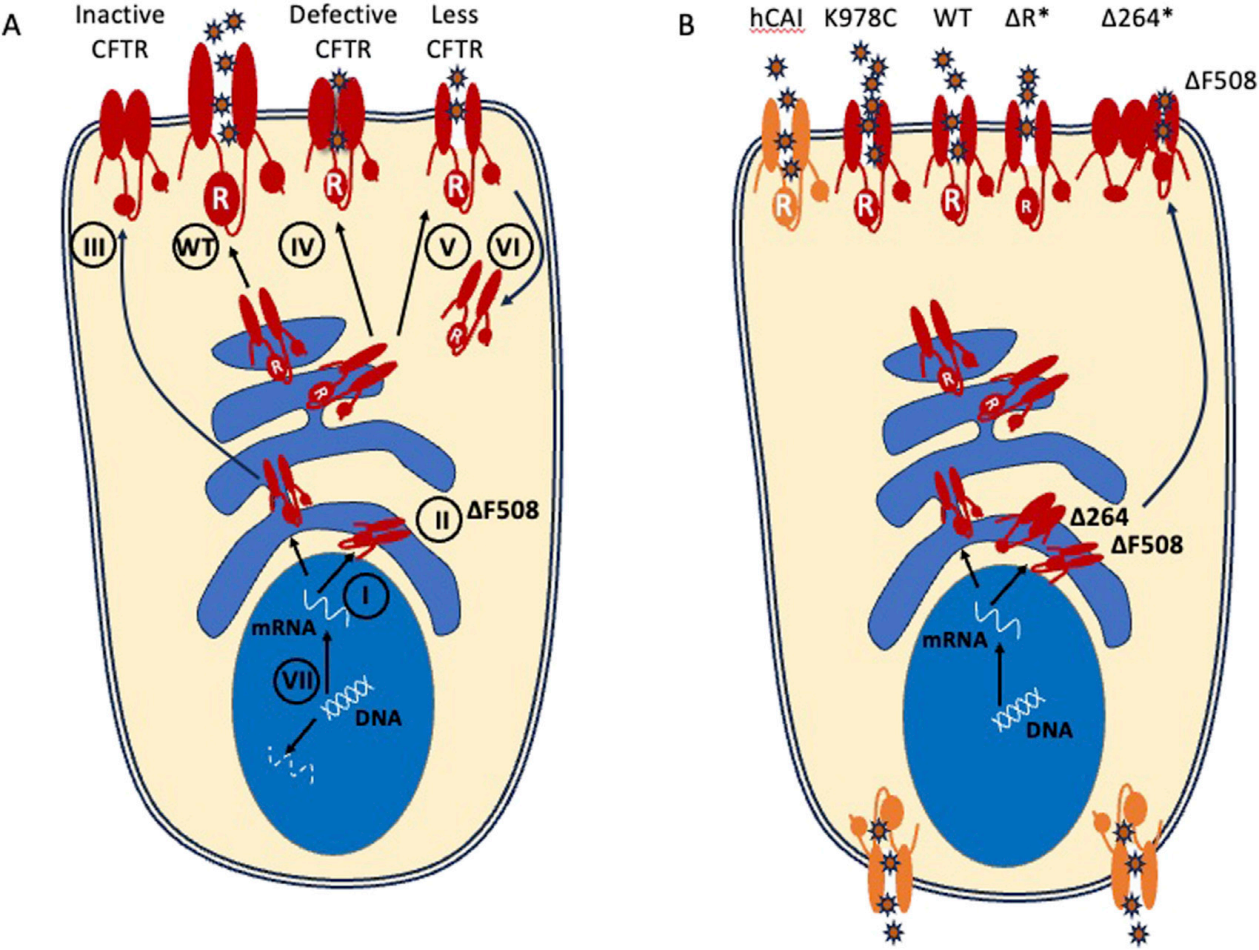
CFTR variant proteins and transgenes and their resulting chloride efflux. **(A)** Class I variants fail to generate protein. Class II variants include the common ΔF508 allele and fail to traffic to the apical surface. Class III variants fail to open at the apical surface. Class IV have decreased conduction properties. Class V and VI have decreased protein stability and increased turnover. Class VII variants have large deletions that do not produce mRNA and are sometimes included as a subtype of Class 1. Small molecule CFTR modulators can only act on mutations which result in stable or full length CFTR protein production that is present in cell (Class II-VI). **(B)** Potential *CFTR* transgenes that have been considered for gene therapy include WT CFTR and gain of function mutants such as (K978C). Codon optimized transgenes (hCAI) may increase protein expression but this has the potential to result in mislocalization. Minigenes (ΔR; Δ264; denoted by asterisks) can fit within AAV packaging constraints but may lose structurally important domains (e.g., Δ264) and should be preclinically tested in multiple variant backgrounds.

CF disease is characterized by a spectrum of severity due to the more than 2000 variants in the *CFTR* gene that result in defects of mRNA production, protein production, trafficking, folding, regulation, conductance, or turnover rate. These variants have been organized into seven classes, summarized in Table 1, based upon the functional consequence of the variant (Figure 1A). PwCF can either have two copies of the same variant (homozygous) or two different disease-causing variants (heterozygous). People with Class I variants lack CFTR protein production resulting from disruption of the *CFTR* coding sequence (e.g., – frameshift mutations). The Class I variant transcripts are either unstable due to premature termination (Class IA) or code for a truncated, dysfunctional protein product (Class IB). Class II variants, including the most common ΔF508, affect the correct trafficking of the protein and result in the loss of CFTR expression at the cell surface. Class III variants include those in which the regulation of the CFTR channel is defective. CFTR variant Classes IV-VI may have a milder CF phenotype with Class IV variants having reduced conduction properties, Class V having decreased stability of mRNA or protein, and Class VI resulting in enhanced CFTR turnover rates (MacDonald et al., 2007). Class VII variants have large deletions that prevent mRNA production, although sometimes these are included as a subtype of Class 1 (DeBoeck and Amaral, 2016). CFTR modulator therapy can improve trafficking and gating of minimal function Class II-VI proteins. However, it is ineffective in Class I/VII patients with no functional protein. These patients represent the most underserved CF population with the most severe form of the disease. Importantly, gene sequencing in new patient cohorts are revealing novel variants and variability in phenotypes with known variants (Gaikwad et al., 2020). This underlies the need for a variant agnostic gene therapy that can deliver a wildtype *CFTR* transgene to lung cells in order to significantly benefit all PwCF.

**TABLE 1:** *CFTR* variant classification and incidence.

Mutation class	Defect	Outcome	Common variants	Relative incidence^a^
I	Protein synthesis	Complete absence of CFTR protein due to premature mRNA termination (nonsense or frame shift mutation)	G542X, W1282X, R553X, 621+G>T	22%
II	Maturation/Processing	Inability of protein to localize to correct cellular location due to abnormal post-translational modifications	F508del, N1303K, A455E	88%
III	Ion channel gating	Decreased activity of protein (chloride channel) in response to ATP due to abnormalities of the nuclear binding fold regions	G551D	6%
IV	Protein conduction	Frequency of flow of ions and channel opening duration are reduced though there is generation of chloride currents on stimulation with cAMP	R117H	6%
V	Reduced amount of functional CFTR	Stability of mRNA and/or mature protein is compromised	A455E	6%
VI	Normal amount of functional CFTR	Enhanced turnover due to C-terminus abnormalities	Q1412X	5%
VII	Protein synthesis	Large deletions with no mRNA production	dele2,3 (21 kb) 1717-1G→A	NA

^a^ People with heterozygous CFTR, variants in two classes are counted twice.

Source: adapted from MacDonald et al. (2007), Rafeeq and Murad (2017), Manfredi et al. (2019).

CF was initially identified and named for the pancreatic phenotype which caused mortality and precluded visible defects in lung function and development of lung disease. Following the emergence of enzyme therapies that resolved the pancreatic insufficiency and nutritional programs for gastrointestinal malabsorption, palliative lung treatments including airway clearance techniques and antibiotic therapy could only extend life expectancy in PwCF to their early 30s (Lopes-Pacheco, 2016). At this time, 90% of patient mortality was still driven by lung disease. Aside from lung transplantation, CFTR modulators represent the current best therapeutic option for patients with relevant CFTR variants, which can benefit from improved transporter function or correction of misfolded transporter proteins. CFTR modulators are not curative but can offer profound disease modifying effects, particularly with the development of the triple combination elexacaftor-tezacaftor-ivafactor (ELX/TEZ/IVA) (Heijerman et al., 2019; Middleton et al., 2019). An interim analysis of ELX/TEZ/IVA demonstrated that the post-treatment annualized rate of death and lung transplantation was 72% and 85% lower than the historical population (Bower et al., 2023) Nevertheless, emerging real-world data also suggest higher rates of adverse events and discontinuation of CFTR modulators compared to clinical trial data. Adverse events described include respiratory (dyspena and chest tightness), gastrointestinal (nausea, abdominal pain and diarrhea) and neuropsychiatric (depression and anxiety), among others (Talwalkar et al., 2017; Dagenais et al., 2020; Shteinberg and Taylor-Cousar, 2020). Although CFTR modulator therapy has presented vast improvements to quality and duration of life for many PwCF, 6%–10% of patients with CF will not benefit from this therapy because they lack eligible CFTR variants (CFF.org). Combined with patients who cannot tolerate CFTR modulators due to adverse events (possibly as high as 20% (Hubert et al., 2017; Burgel et al., 2019) more than 20%–25% of PwCF remain underserved with lung transplantation and/or quality of end-of-life care as their only options (Mercier et al., 2021; Hisert et al., 2023). Patients who are ineligible or unable to tolerate CFTR modulatory therapy present a high unmet need. Given the monogenic molecular cause of CF, and evidence that lung disease is the primary cause of morbidity with current therapeutics, there is great potential for a lung directed gene therapy to improve quality of life and extend lifespan in these patients.

## 3 How much CFTR is needed?

Gene therapies are composed of vectors that can bind and enter, or transduce, specific target cells and deliver wildtype genes to generate functional protein. To understand the level of airway transduction necessary for a *CFTR* gene therapy to rescue lung function, several groups have measured the amount of *CFTR* expressing cells required to restore function in a CF airway model *in vitro*. *In vitro* models, often described as the Air-Liquid Interface (ALI) culture method, use primary human bronchial epithelial cells (HBECs) derived from bronchial brushing or transplanted CF lungs, and are considered the gold standard to measure CFTR function from CFTR variants and to validate the efficacy of CFTR therapeutics preclinically (Awatade et al., 2018). HBEC cultures form tight junctions, which allows the use of electrophysiological measurement techniques (e.g., Ussing chambers) to assess airway conductance and ion transport. Rescue of CFTR-mediated ion transport in CF HBECs has translated to clinically relevant lung function improvements (Keating et al., 2018). For example, the CFTR modulator lumacaftor/ivacaftor restores CFTR function to approximately 14% of normal levels *in vitro* (VanGoor et al., 2011). This resulted in sustained 2%–4% improvement in FEV1 and 30% reduction in exacerbations (Wainwright et al., 2015). Cell mixing experiments using genetically engineered cell lines as well as patient-derived primary human bronchial epithelial cells have demonstrated that epithelial sheets containing 6%–10% WT CFTR-expressing cells generated functional activity similar to epithelial sheets containing 100% WT CFTR-expressing cells (Johnson et al., 1992; Dannhoffer et al., 2009). Gene transfer studies using an adenoviral delivery vector that transduced only 20% of CF HBECs fully rescued ion transport with a WT *CFTR* transgene (Excoffon et al., 2009), and a similar study using a human parainfluenza viral vector demonstrated normal CFTR activity with 25% of the epithelium transduced (Zhang L et al., 2009).

**FIGURE 2:**
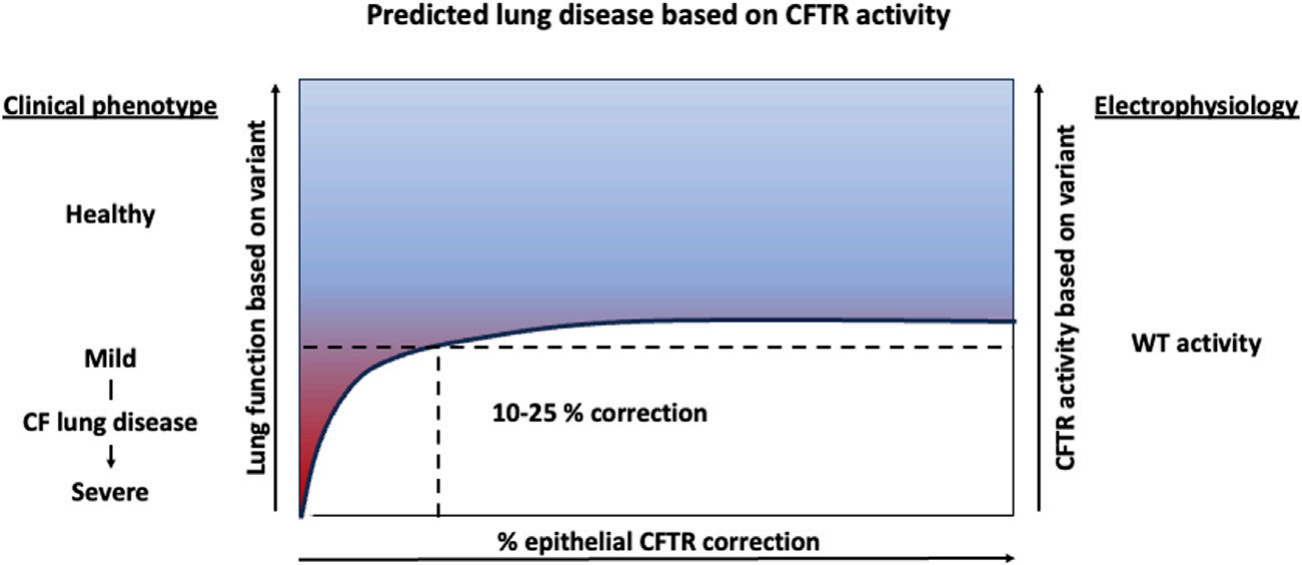
Predicted lung function improvement with increasing CFTR activity. Clinical data suggest 5%–10% CFTR activity results in severe lung disease and CFTR rescue experiments in human bronchial epithelial cell cultures suggests that 10%–25% transduced epithelial cells can restore near WT electrophysiology activity *in vitro*.

Further methods of deriving a relationship between CFTR expression, function and disease severity include utilization of *in vitro* airway models and clinical sequencing of patient samples and lung function data. Recently, a group edited human bronchial epithelial cells (HBECs) to express patient specific *CFTR* variants and measured the resulting CFTR protein activity using electrophysiology assays. In this study, patient variants associated with full expressivity of CF disease (meaning they developed pancreatic, lung and sweat chloride phenotypes) were demonstrated to have less than 10% WT CFTR protein activity *in vitro* (McCague et al., 2019). Additionally, PwCF having a common splicing variant (3272-26A>G/F508del) were found to express approximately 5% of the normal levels of wildtype (WT)-*CFTR* mRNA and have milder CF disease (Ramalho et al., 2012). This suggests that having only 5% WT-CFTR is sufficient to ameliorate severe disease phenotypes. Studies such as these have established the bar of 5%–10% normal CFTR activity to achieve significant health benefit in PwCF. Similarly, a study found a non-linear, logarithmic relationship between CFTR activity (predicted by variant status) and lung function, suggesting that even small increases in functional CFTR would provide vast improvement in people with severe lung disease. These data again suggest that low levels of airway epithelial cell transduction can improve lung function in a CF airway, with a target of 5%–10% transduction ameliorating severe disease and a target of 10%–25% transduction restoring lung function in PwCF (Figure 2).

## 4 Where and how does CFTR function?

In order to target the right cells with a CFTR gene therapy, we need to understand where and how CFTR normally functions. The upper airway is primarily comprised of basal cells, which cover the basolateral membrane and act as progenitor cells for all airway cell types, along with secretory cells that produce mucus to trap pathogens, and ciliated cells, which have hairlike projections that sweep mucus and its attached pathogens from the airway (mucociliary clearance). In the lung, defective CFTR function results in decreased anion secretion (chloride and bicarbonate) ((Rich et al., 1990)). The change in osmolarity leads to the removal of water from the airway secretions, which become relatively dehydrated and viscous making them difficult to remove by either mucociliary clearance or by cough. Subsequently, the retained secretions invite opportunistic bacterial and fungal infections. Work from CF transgenic animal models has elucidated CF lung pathogenesis (Sun et al., 2010). For example, newborn *CFTR−/−* pigs display early congenital airway abnormalities, including lowering of pH which hinders the bacteria killing function of airway surface liquid, defects in mucus detachment from submucosal glands, and early infection that precedes inflammation and remodeling (Stoltz et al., 2015).

**FIGURE 3:**
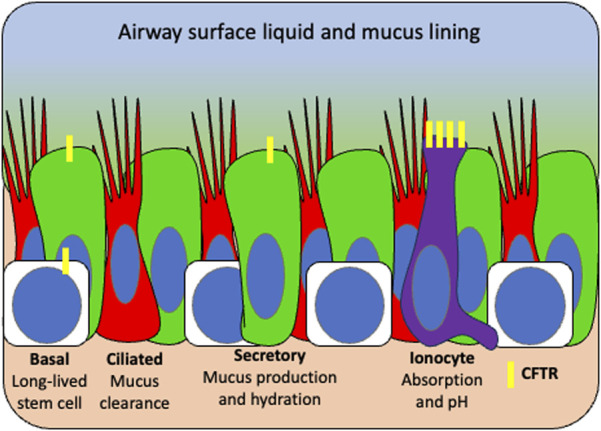
Surface airway target cell types for CFTR gene therapy based on distribution of functional tasks. Basal stem cells (white) repopulate the airway and express low levels of CFTR (yellow). Secretory cells (green) produce mucus and antimicrobial peptides and also express low levels of CFTR which functions to hydrate airway surface liquid. Ciliated cells (red) sweep hydrated mucus from the airway and produce little to no CFTR. Ionocytes (purple) express high CFTR which functions in pH balance and is thought to mediate bacterial killing. Given that multiple cell types perform CFTR functional tasks, basal stem cells would be the ideal target for a durable gene therapy so that CFTR can be retained in all daughter cells.

The complex tasks of mucus production and hydration, bacteria killing and mucociliary clearance are distributed among specialized airway cell types (Figure 3). Understanding which cells can utilize CFTR to restore these functions is critical to understanding which cell should be targeted with a gene therapy. In addition to high expression in the submucosal glands, it is now appreciated that airway secretory and basal cells express the majority of *CFTR* in the surface airway epithelium but at low levels (∼1-2 mRNA transcripts) per cell (Okuda et al., 2021). The recently discovered pulmonary ionocyte, despite being only 1%–2% of the airway epithelium, expresses the remaining *CFTR* (>30 mRNA transcripts per cell) (Montoro et al., 2018; Plasschaert et al., 2018). In order to elucidate the function of CFTR in these distinct cell types, transgenic ferrets were used to ablate ionocytes thus demonstrating their role in airway surface liquid (ASL) absorption and secretion and mucus viscosity (Yuan et al., 2023). It was further revealed through manipulation of ionocyte abundance and expression of cell type specific ion channels in HBECs *in vitro* that CFTR plays distinct roles in ASL absorption and secretion, in ionocytes and secretory cells, respectively (Lei et al., 2023). Finally, using single cell electrophysiology experiments, it was shown that ionocytes regulate ASL pH through an anion exchanger that secretes bicarbonate into the lumen, thus increasing the pH (Luan et al., 2024). These data demonstrate that distinct, specialized cell types utilize CFTR to maintain ion homeostasis in order to modulate ASL hydration, mucus viscosity, and pH.

Ideally, a lung-directed gene therapy would target all airway cells that endogenously express *CFTR* in both the surface airway epithelium and submucosal glands. It is unclear whether an inhaled gene therapy would reach distal *CFTR*-high serous cells in submucosal glands; however, proximally located progenitor cells may be more accessible (Jiang and Engelhardt, 1998; Anderson et al., 2017). There may be benefit in transducing some surface airway epithelial cells in order to disrupt the mucus barrier for repeat dosing, although it is still unclear whether all airway cells can use CFTR effectively (Ostrowski et al., 2007). The best preclinical model for CFTR restoration and function is the HBEC culture model which is composed of the surface airway epithelial cells but lacks submucosal gland cells. It's critical to remember that CFTR modulators work on existing endogenous protein; therefore, HBEC models can predict how a modulator will work on all CFTR expressing cells in the body. However, in gene therapy studies, *in vitro* models should be combined with animal biodistribution studies to determine which airway cell types are reached *in vivo*. While efficacy studies *in vivo* are also highly desirable, there are few CF animal models and gene therapy vectors can be species specific preventing their translatability (Excoffon et al., 2009; Steines et al., 2016). Nebulization of CF pigs with a pig tropic vector expressing *CFTR* demonstrated that delivery to the surface epithelial cells of the trachea demonstrated improvements in ASL pH and partial rescue of bacteria killing (Steines et al., 2016). These data, along with the partial transduction of epithelial sheets *in vitro,* hint that not all cells need to be transduced throughout the lung to achieve therapeutic benefit.

(Zhang et al., 1998; Ostedgaard et al., 2002) The durability of the gene therapy will be determined by the percentage and turnover of the cell types transduced. Studies in mice suggest secretory cells turnover every 11 days (Watson et al., 2015). Extrapolating this to humans, a gene therapy that targets only differentiated cells, including secretory cells and ionocytes, may need redosing on a yearly basis but one that reaches basal cells as well may not need redosing for 5–10 years. The CF lung environment may also influence turnover (Carraro et al., 2021). Ultimately, a durable gene therapy would contain either 1) a highly lung tropic vector that is non-immunogenic so that it can be readministered without eliciting an immune response or 2) an editing or integrating cargo targeted to basal cells which can repopulate the airway epithelium during turnover, thus expanding and retaining functional *CFTR* long term.

Finally, the genetic cargo will influence the expression and function of a gene therapy. This review focuses on a gene transfer strategy rather than an editing strategy. To design a cargo for gene transfer, the sequence of the *CFTR* transgene is strongly considered. An obvious choice would be to express the wildtype *CFTR* transgene, but not all gene delivery platforms can deliver a transgene of this size (∼4.5 kb). To overcome this limitation for AAV-based viral delivery where transgenes must be limited to <4.4 kb, a functional *CFTR* minigene has been generated from deletions in the regulatory (R) domain (Zhang et al., 1998; Ostedgaard et al., 2002). Elements such as reduced CpG content in the transgene are also a potential consideration, as it may decrease the risk for an immune response (Ronzitti et al., 2020). Furthermore, gain of function *CFTR* variants have been shown to further enhance efficacy (Woodall et al., 2023). The promoter included to express the *CFTR* transgene can be cell-type specific but given the plethora of cell types expressing CFTR in the lung, this may limit its functional rescue. Alternatively, a strong, ubiquitous promoter can boost expression, thus improving efficacy, but may push expression beyond physiologically-relevant levels which can result in diminished pharmacology and safety considerations through the mis-localization of CFTR (Farmen et al., 2005). The promoter is also included in the cargo packaging capacity, notably limiting certain vectors such as AAV. Although there are clear benefits to expressing the wild type *CFTR* transgene to minimize the risk of novel epitope formation and deliver clear therapeutic benefit, further safety and efficacy benefits may emerge following optimization studies, and the field has yet to fully align on the best therapeutic approach (further reviewed in Section 5.1).

## 5 Gene therapy for cystic fibrosis

Gene therapies offer disease modulation of a misfolded protein with multiple agents acting as both correctors and potentiators of CFTR. CF has historically been considered a strong candidate for gene therapy approaches since the genetic cause was identified in 1989 (Riordan et al., 1989; Wagner et al., 1999). Furthermore, the lung is a seemingly accessible organ for an inhaled or bronchoscopically-delivered gene therapy which would have the potential to address the most severe manifestation of disease and prolong lifespan in PwCF. However, over 20 clinical trials have attempted airway delivery of genetic material through modified or synthetic vectors to lung epithelial cells (Alton et al., 2016) and failed to achieve therapeutically relevant levels of expression. These failures are largely attributed to a number of factors, including insufficient potency, a lack of relevant cell specificity, and CF mucosal and immune barriers. Here, we briefly summarize the field of *CFTR* gene therapy with particular focus on the advantages and limitations of advanced gene delivery vectors (Table 2).

**TABLE 2:** Characteristics of viral vectors used for CFTR gene therapy.

	AAV	LNP	HBoV1	Lentivirus	HSV1
Ability to package full length CFTR?	No	Yes	Yes	Yes	Yes
Cargo	ssDNA	mRNA	ssDNA	RNA	dsDNA
Expression	Stable, non-integrating	transient	Stable, non-integrating	Stable, integrating	Stable, non-integrating
Immunogenicity	Low	High	Low	Mod-high	Low
Redosing Potential	Low	++	++	-	+
Evolved Airway tropism	Serotype dependent	Historically low without targeting peptides	High	Historically low due to location of receptors	Low

### 5.1 Viral delivery vectors

**FIGURE 4:**
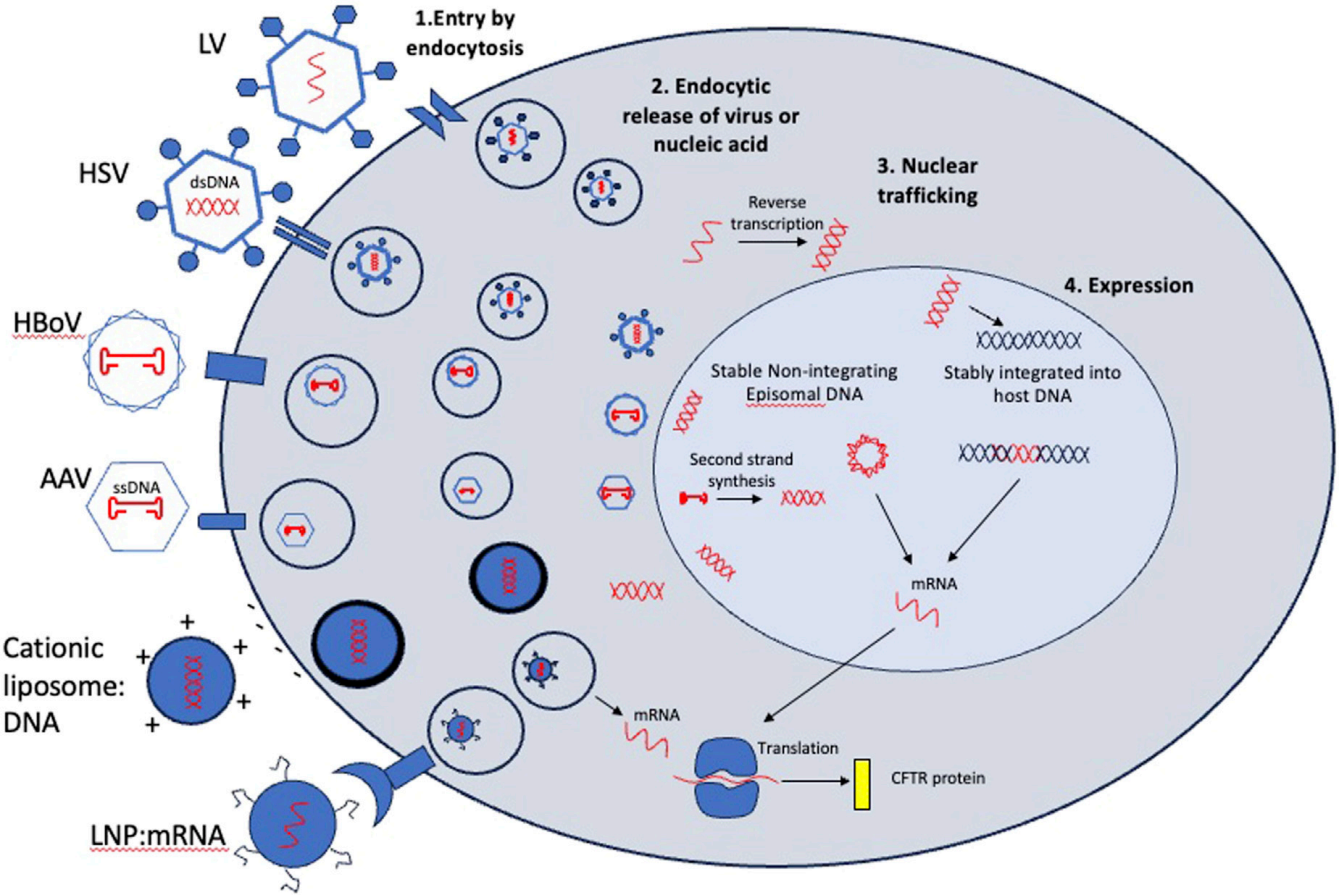
Entry mechanisms and post-entry processing of viral and non-viral vectors considered for CFTR gene therapy. In step 1, vectors bind to cell surface receptors or molecules or fuse directly to the plasma membrane (Cationic liposome:DNA) and are endocytosed. In Step 2, viral particles or nucleic acids are released into the cytoplasm. This is a rate limiting step for some viruses which need an expression enhancer such as doxorubicin to improve endocytic release and nuclear trafficking. In step 3, vectors containing DNA are trafficked to the nucleus while mRNA cargos are translated in the cytoplasm. In step 4, some cargos are integrated into the host chromosome (lentivirus cargos) while others remain episomal. Integrated cargos will be retained after every division while episomal cargos will be diluted out with cell division. mRNA cargos will be transient in transduced cell.

Viruses have evolved over hundreds of millions of years to effectively deliver genetic payloads, which is conceptually similar to gene therapy. Therapeutically, these viruses have been engineered to serve as delivery vectors through removal of all endogenous replicative machinery, producing recombinant versions of the viral capsid protein shell in a tightly controlled manufacturing process that can be packaged with a specific therapeutic transgene. Their tropism or ability to transduce different cell types is determined by their unique capsid or envelope proteins which bind to cell receptors or cell surface molecules. Most vectors use endocytosis for cell entry, and endocytic release and nuclear trafficking for expression of genetic cargo. However, their nucleic acid cargo and method of expression can vary greatly (Figure 4).

From 1993–2001, nine clinical trials used adenovirus as a delivery vector for full length *CFTR* DNA. Unfortunately, transduction was minimal and historical endpoints of improvements in membrane potential difference (CFTR function) and interleukin levels (inflammation) were not consistently met across trials (Cooney et al., 2018)). A quantitative measurement of gene transfer in one study reported 5% of endogenous *CFTR* levels were delivered by transgene 3 days post adenoviral dosing; however, exogenous *CFTR* returned to baseline 30 days later, and could not be detected 3 days following a third dose (Harvey et al., 1999). Failure of adenovirus to express sufficient and sustained *CFTR* in human airway epithelium has been attributed to high immunogenicity to the viral vector and inability of the vector to access basolaterally-located viral receptors from the inhaled delivery route.

To overcome the high immunogenicity and poor tropism of adenovirus for apical entry, non-pathogenic adeno-associated virus (AAV) subsequently became the preferred gene therapy vector to deliver *CFTR* (Wagner et al., 1998). AAV2 was shown to transduce polarized airway epithelia (Flotte et al., 1994). However, gene transfer in the clinic was again transient as demonstrated by a Phase IIb study in which vector genome copies decreased from 0.6 copies per brushed airway cell at day 14 to 0.1 copies at day 30. No vector was detected at 90 days (Wagner et al., 2002). Notably, these studies measured vector genome (DNA) rather than transgene expression (mRNA) and likely overestimated transduction since post-entry barriers of virion processing following infection appear to limit nuclear translocation and mRNA expression (Figure 4; steps 2–4). Although AAV2-*CFTR* delivery was deemed to be safe and well tolerated by the patient, it ultimately failed to demonstrate significant functional efficacy and sustained disease modification. Additional naturally occurring AAVs have been identified with improved tropism for various airway cell types. For example, AAV5 showed a 10-fold improvement in transduction over AAV2 in non-human primate lung (Fischer et al., 2007). Current programs are using new AAVs with capsids evolved from mutagenesis or recombination and selected for better airway tropism (Excoffon et al., 2009; Steines et al., 2016).

AAVs continue to be a preferred vector for gene delivery *in vivo* because they have low immunogenicity and their cargo is expressed from episomes, rather than integrating into the host chromosomes (Table 2; Figure 4). However, a major limitation for the use of AAVs as a *CFTR* delivery vector is a small genome packaging capacity. AAVs can package ∼4.7 kilobases of DNA with ∼3 kb being reserved for AAV machinery, leaving <4.4 kb for therapeutic cargo. A strong promoter combined with the full length *CFTR* coding sequence (∼4.5 kb) exceeds this. The early AAV2 gene therapies utilized the endogenous AAV2 promoter located within the inverted terminal repeats (ITRs) of the AAV machinery. Subsequently it was determined that low expression and variable functional efficacy could be attributed to this feature, in addition to poor transduction by the capsid. This has remained a major barrier to efficient *CFTR* expression from AAV-based vectors. To overcome this limitation, work has been done to generate a functional *CFTR* minigene (Zhang et al., 1998; Ostedgaard et al., 2002). Of note, a variant deleting the first 4 transmembrane segments of TMD1 (Δ264) was shown to rescue CF human airway cultures expressing the most prevalent Class 2 variant (ΔF508). However, it was subsequently shown to rescue via transcomplementation and trafficking of endogenous mutant protein and does not conduct chloride on its own (Cebotaru et al., 2013) (Figure 1B). This demonstrates the importance of testing AAV-*CFTR* gene therapies in different CF backgrounds, particularly with Class 1 variants, which lack endogenous protein.

Additional *CFTR* minigenes have been generated from deletions in the regulatory (R) domain. One such has shown functional restoration in HBECs and preclinical species (Ostedgaard et al., 2005); Figure 1B) and is currently being tested in a clinical trial with a capsid evolved for non-human primate airway tropism (AEROW; NCT05248230). The R domain is unique for CFTR as it is not present in other ABC transporters. Phosphorylation sites are highly conserved across species and impact CFTR gating, localization in the plasma membrane and interaction with additional ion channels. One deletion, Δ708-759, functioned most similarly to wild type CFTR in cell-based assays with a non-significant reduced chloride efflux compared to wildtype CFTR, potentially due to a reduction in phosphorylation sites (Ostedgaard et al., 2002). Binding sites in the R domain have been shown to be important for an interaction with 14-3-3, an adaptor protein which increases the level of CFTR in the plasma membrane (Liang et al., 2012). Variants found in the CF database (www.genet.sickkids.on.ca), including S753R, V754M, and R766M, have reduced binding to 14-3-3 compared to the wildtype residues (Stevers et al., 2016). Finally, phosphorylation of the R domain increases binding to the SLC26 family of chloride/biocarbonate transporters which stimulate CFTR activity in epithelial cells (Ko et al., 2004). These data warrant development of a vector that can deliver full length *CFTR* retaining all potentially important binding domains. Furthermore, *CFTRΔR* (708–759) only removes 156 nucleotides from the *CFTR* gene (Ostedgaard et al., 2002). While *CFTR* minigenes facilitate transgene packaging into AAV capsids, they are still sufficiently large to cause instability in the 5′region of the AAV genome (Kapranov et al., 2012). These challenges suggest that vectors that could carry a full length *CFTR* transgene (larger payload capacity) would be preferred candidates for CF therapeutic interventions.

Novel vectors are being explored that can package full length *CFTR* and traditional vectors are also being modified by pseudotyping, or the process of engineering the capsid or envelope proteins to alter tropism. Lentiviral vectors have traditionally shown poor tropism for airway without physical or chemical disruption to the tight junctions in order to allow access to the basolateral receptors (McCarron et al., 2021). However, new lentiviral vectors pseudotyped with the Sendai virus envelope proteins F and HN has been shown to transduce intact airway epithelium via the apical domain (Alton et al., 2017). While lentivirus has a large packaging capacity, it is an integrating virus as opposed to the non-integrating, episomal AAV (Figure 4). Integrating viruses have the potential to introduce genomic instability or oncogenicity and until now, have primarily been used for *ex vivo* transduction of cell therapies in which the integration site can be analyzed and off target effects are minimized. Non-integrating lentiviral vectors are being engineered without viral integrase to circumvent this (Milone and O’Doherty, 2018). It is worth nothing that lentivirus captures membrane proteins from cell lines during the manufacturing process which can increase immunogenicity.

Herpes-simplex virus 1 (HSV1), with a packaging capacity of >30 kb, can deliver two copies of *CFTR* DNA, and has a non-integrating episome like AAV (Epstein et al., 2005). HSV1 traditionally infects epithelial cells and sensory neurons although it is not known to be an airway tropic virus. Unlike AAV, natural HSV is pathogenic, however recombinant vectors are non-replicative. Modified HSV for gene therapy is currently being tested in a clinical program for Dystrophic Epidermolysis Bullosa for keratinocyte transduction when applied topically (Epstein and Haag-Molkenteller, 2023).

Finally, the human bocavirus type 1 (HBoV1) is a promising candidate for *CFTR* gene therapy as its natural host cell is polarized airway epithelia. Native pathogenicity of this virus is still being elucidated as it often occurs as a co-infection with additional respiratory pathogens ((Rajme-López, 2024). Importantly, both HBoV1 and AAV are parvoviruses and through pseudotying, the HBoV1 capsid has been engineered to package the AAV2 genome expression elements (Figure 4). Therefore, like AAV, the HBoV1 therapeutic cargo remains episomal and does not integrate into the host genome. This derisks HBoV1 as a gene delivery vector as the safety of gene delivery through AAV expression cassettes has largely been established over many clinical trials, particularly with lung-directed delivery (https://clinicaltrials.gov/). The HBoV1 genome packaging capacity is 5.5 kb expanding that of AAV and allowing for inclusion of a full length, wildtype *CFTR* along with a strong promoter. HBoV1 has been shown to transduce animal models *in vivo* and shows significant improvements over AAV2 apical transduction in polarized airway epithelial cells *in vitro*. Furthermore, HBoV1-*CFTR* can rescue chloride efflux in CF HBECs (Yan et al., 2013; Yan et al., 2017). Additionally, this vector has demonstrated re-infection in young children (Meriluoto et al., 2012) suggesting it has the potential to be readministered in contrast to current AAVs, which is necessary for the durability of non-integrating vectors.

With larger packaging capacity, there may also be utility in expressing gain of function (GOF) *CFTR* transgenes that are able to allow for more chloride efflux per channel. One such GOF mutation, K987C increases open probably of the channel regardless of ATP binding ((Wang et al., 2010); Figure 1B). A further effort to increase expression of therapeutic protein delivered by gene therapies is through codon optimization (Mauro and Chappell, 2014). While this does not change the amino acid sequence, it can optimize the rate and level of protein generated. One study has demonstrated drawbacks of codon optimization for *CFTR* gene therapy as it led to mislocalization of both the wildtype CFTR and the GOF CFTR (K987C), potentially through high expression forcing it to expand beyond the apical membrane (Woodall et al., 2023). This resulted in symmetrical transport of anions rather than the polarized vectoral transport across the apical membrane which drives hydration. While these experiments have been performed *in vitro* and the physiological implications on mucociliary transport are not clear, it suggests that restoring a moderate amount that most closely mimics the low levels seen in the majority of airway epithelial cells could be important for proper function. The CFTR cargos that have been proposed for various delivery vectors are schematized in Figure 1B.

### 5.2 Non-viral vectors

Finally, nonviral delivery methods offer an alternative to viral delivery and do not include payload size constraints. One such method utilizes cationic liposome:DNA complexes that fuse with the negatively charged plasma membrane for direct entry into the cell (Figure 4). In several clinical trials testing nebulization of cationic lipid:*CFTR* complexes, early evidence for partial correction of CFTR-mediated chloride transport was measured by changes in transepithelial potential difference. However, expression and duration of improvement were transient even with repeat administration (Cooney et al., 2018). It was speculated that early derivations of lipid:DNA complexes were inefficient due to an unexpected majority of the lipid complex being endocytosed leading to retention of DNA in perinuclear vesicles (Zabner et al., 1995). Subsequently, more efficient lipoplexes were synthesized. A Phase IIb trial testing monthly repeated nebulization of a lipid:*CFTR* complex stabilized lung function over the course of 1 year (Alton et al., 2015), demonstrating one of the most meaningful outcomes to date as CF patient lung function declines at ∼1–2% per year (Leung et al., 2020). However, an improvement in lung function has proven possible with CFTR modulator therapy and is still the primary efficacy endpoint.

Lipid nanoparticle (LNP) delivery of *CFTR* mRNA offers additional advantages by not requiring nuclear translocation, a rate limiting factor in DNA delivery (Figure 4). Historically, LNPs have shown high immunogenicity and low transduction of airway epithelium. A recently tested LNP (MRT-5005) demonstrated a 1500-fold increase of transgene expression over endogenous *CFTR* in preclinical species, rescue of CF human airway epithelial cells with an optimized *CFTR* mRNA, and an ability to be redosed resulting in sustained expression, an essential requirement for a transient mRNA gene therapy. While early data in a Phase 1/2 trial indicated an improvement in lung function tests 8 days following a single dose of nebulized MRT-5005, that improvement was not sustained at 30 days and weekly dosing did not result in meaningful improvement in lung function over placebo after 12 months. Despite strong transduction and gene transfer in preclinical species (Robinson et al., 2018), it was reported that LNPs have failed to reproducibly deliver mRNA to polarized human airway cultures (Rowe et al., 2023). An additional LNP-delivered mRNA (VX-522) is enrolling patients that are ineligible for modulators in a Phase1/2 trial (NCT05668741). The mechanisms of entry and expression (Figure 4) as well as the advantages and disadvantages (Table 2) of delivery vectors should be used to prioritize the most promising gene therapy programs for PwCF.

### 5.3 Lung gene therapy delivery devices and adjuvants

Early lung-directed gene therapies were applied as a liquid bolus to the epithelium by nasal infusion or bronchoscope instillation (Knowles et al., 1995; Zuckerman et al., 1999). In more recent years, aerosolization has been optimized in order to generate smaller droplets that can reach more distal regions of the lung via a nebulizer (Pleasants and Hess, 2018), and these have been applied to test both viral and non-viral gene therapies for CF (Aitken et al., 2001; Davies et al., 2014). Despite improvements in penetration via formulation of the delivery vector, there are additional barriers such as the mucus obstruction in CF lung disease and inability to access underlying basal progenitor cells or basolaterally located receptors as discussed above. Chemical conditioning reagents such as lysophosphatidylcholine (LPC) and polidocanol as well as physical disruption of lung barriers have been utilized to great effect in increasing transduction in animal models but are limited in their clinical translation due to safety concerns, particularly the risk of allowing access of antibiotic resistant bacteria to the circulation (McCarron et al., 2023).

Even with successful transduction, another mechanism to overcome is subtherapeutic expression of *CFTR* transgene. Proteasome inhibitors such as doxorubicin have shown an enhancement of gene transfer and expression in the lung when combined with viral and non-viral vectors. While the mechanism is imprecisely understood, potential improvements may be due to improved virus degradation and genome uncoating, improved nuclear translocation of cargo, improved second strand synthesis and reduced degradation of recombinant protein (Figure 4; steps 2–4). Doxorubicin has been shown to enhance gene expression in polarized airway epithelia following delivery with AAV2 and AAV5 *in vitro* and delivery with HBoV1 and a lipid:DNA complex *in vivo* (Yan et al., 2004; Yan et al., 2017; Griesenbach et al., 2009). Furthermore, it has shown enhancement with non-airway cell types, and in additional species, including NHP, suggesting this is a more common, universal mechanism (Zhang T et al., 2009; Zhang et al., 2012; Gong et al., 2021). While enhanced expression was demonstrated both in number of cells expressing the transgene as well as higher expression per cell, data suggest doxorubicin does not alter vector tropism and likely increases the transgene expression in transduced cells to detectable levels (Zhang T et al., 2009). Finally, doxorubicin was shown to induce more rapid transgene expression, which is critical for patient improvement and successful early endpoints in clinical trials and may allow for a reduced viral dose as demonstrated by a similar level of transgene expression when combined with doxorubicin at a 10-fold lower dose of AAV (Gong et al., 2021). The additional mechanism of doxorubicin as a DNA intercalating agent poses a concern for using it as a gene expression enhancer. However, the dosing used to demonstrate gene enhancement was localized and more than 1000-fold below the doses associated with dose limiting findings in a Phase I/II lung cancer trial evaluating nebulized doxorubicin when co-administered with a standard chemotherapy and radiation treatment regimen (Otterson et al., 2010; Gong et al., 2021). Thus, most investigators anticipate if doxorubicin is used in CF gene therapy trials, it would be: 1) inhaled, restricting exposure to the airway 2) administered at very low dose compared to inhaled cancer therapy dosing 3) likely be a one-time dose.

An additional barrier to gene delivery is an immune response which can be somewhat mitigated through coadministration of corticosteroids such as prednisone. Steroid treatment can be initiated prior to gene therapy dosing, concurrently, or subsequently, in response to heightened liver enzymes which primarily occurs with systemic delivery. If prednisone will be administered in the clinical trial, it should be tested in preclinical safety studies as well. While there is some evidence that prophylactic immunosuppression with corticosteroids increases transduction and transgene expression from a systemically delivered gene therapy in preclinical species (Wang L et al., 2022), it is unclear whether the same effect is true for lung-delivered gene therapies. Pulmonary exacerbations are a concern for PwCF (see Section 6.3) and may be initiated by an exogenous gene delivery vector. However, it is worth noting that the benefit of prescribed corticosteroids for pulmonary exacerbations is under question (Muirhead et al., 2021).

Finally, it is possible that a *CFTR* gene therapy will be tested in combination with existing CFTR modulators, either due to an ethical concern of taking patients off modulators or to achieve an additive effect via different therapeutic modalities and mechanisms. Because CFTR modulators act systemically, and a lung-directed gene therapy would only ameliorate the lung disease, patients with appropriate variants may want to continue with modulator therapy to manage systemic symptoms. Additionally, potentiators such as Genestein and Ivacaftor may provide enhanced benefit by increasing the open probability and chloride efflux through an exogenously delivered CFTR therapeutic protein (Mukherjee et al., 2022). Theoretically, this can improve shortcomings in transduction and expression by optimizing the activity of the delivered transgene. Importantly, it is necessary to test the synergy between a *CFTR* gene therapy and CFTR modulators in preclinical studies, particularly in the case of *CFTR* minigenes, to confirm that they can bind and modulate the novel protein.

## 6 CF clinical trials and biomarkers

Due to improvements in standard of care, including CFTR modulators, there are now more adults with CF than children, and it is predicted that the new era of modulators will increase lifespan in PwCF beyond the fifth decade of life (Allen et al., 2023). In PROMISE, a real world, post approval observational study, 6-month data demonstrated improvements in FEV1, body mass index, quality of life, and sweat chloride concentration (Nichols et al., 2021b). Therefore, the development of modulators has resulted in a CF community where a large population exhibit mild, possibly stable, lung disease and a smaller population have no disease-modifying therapy or cannot tolerate the available therapies. Future therapeutics will be challenged with demonstrating efficacy in people currently on modulators with marginal windows for clinical improvements. CF gene therapies have shown promising stabilization or temporary improvement of lung function clinical endpoints (Alton et al., 2016; Rowe et al., 2023) but have not demonstrated a sustained improvement in lung function; and going forward, a growing number of gene therapy programs are competing for a small pool of PwCF for their clinical trials. This necessitates greater prioritization of preclinical programs using various gene therapy vectors (Table 2) and novel, sensitive endpoints for lung function.

### 6.1 CFTR function

**FIGURE 5:**
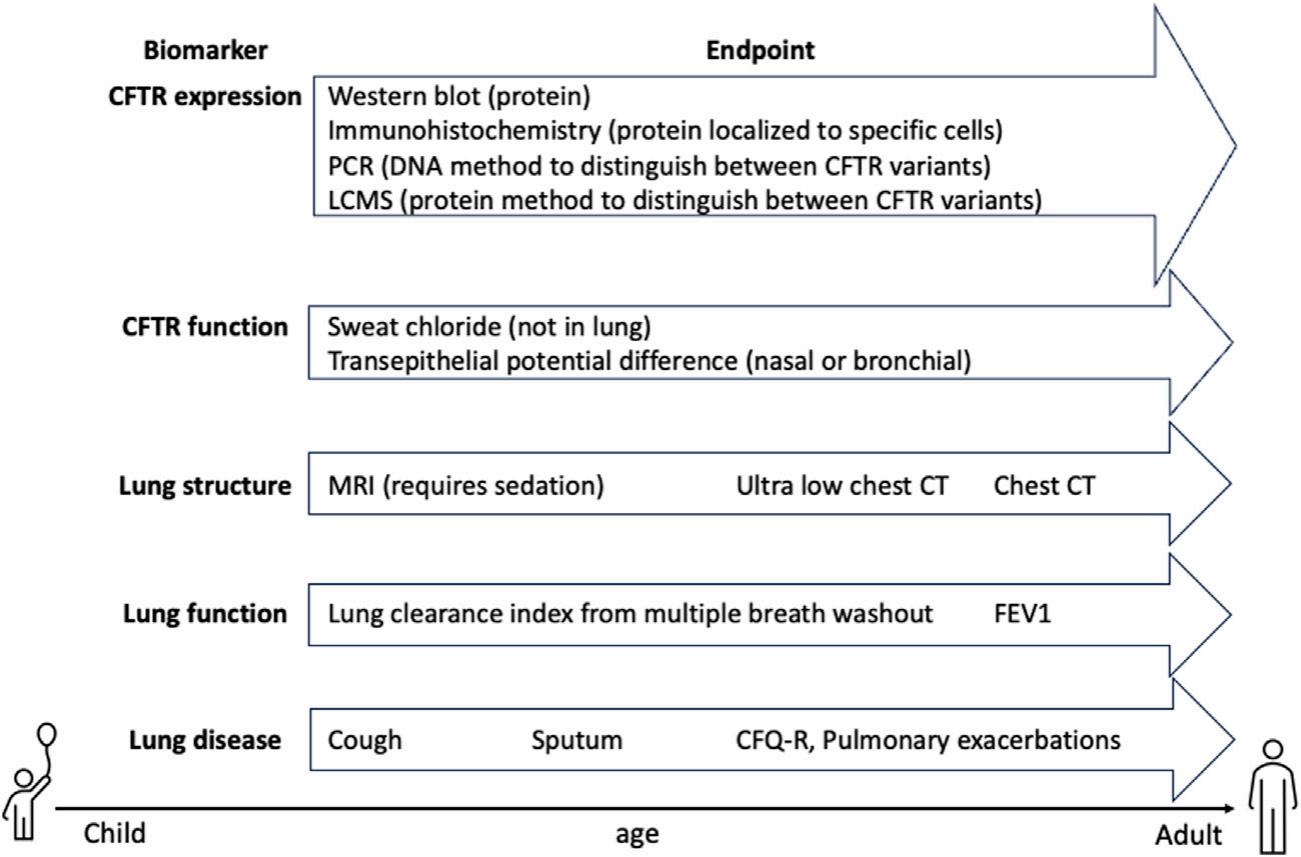
CFTR clinical trial biomarkers. Pharmacodynamic endpoints that detect CFTR expression and function are critical for ensuring delivery of CFTR to relevant cell types. Lung structure, function and disease endpoints are critical for determining whether PwCF will benefit from therapy and thus, need to be sensitive enough to measure changes in mild disease or in combination with modulators. Furthermore, endpoints that can measure early disease and can be analyzed in young PwCF are important for expanding trials to pediatric populations.

Several gene therapies have shown an early improvement of lung function over placebo that disappears over time (Alton et al., 2015; Rowe et al., 2023). This raises the question of whether the initial response to treatment was unrelated to CFTR restoration such as an acute heightened immune or mucociliary clearance response. Alternatively, it could be evidence of lack of durability of the gene therapy resulting in loss of CFTR over time. For this reason, expanding endpoints in CF clinical trials include measurements of CFTR protein expression and function as well as lung structure and lung function and finally, lung disease (Figure 5). Theoretically, an improvement in CFTR function should result in improvements in lung function and decrease in lung disease through the mechanisms described in Figure 3. Sweat chloride has been a primary, non-invasive readout of global CFTR function, although it is not related to lung health. It is debatable how well sweat chloride correlates with spirometry measurements of lung function. Analysis of 8 clinical trials testing the effect of Ivacaftor on gating variants demonstrated a correlation between sweat chloride and ppFEV1 across all studies but not in any individual study. These data suggest sweat chloride can be a predictive pharmacodynamic biomarker of lung function improvement across a population but not for individuals (Fidler et al., 2017). Additionally, in a 6-month study of triple combination modulator treatment, each 10-point decrease in sweat chloride was associated with a 0.89 point increase in ppFEV1 (Nichols et al., 2021a). It is unlikely that an inhaled *CFTR* gene therapy would access the sweat glands; therefore, additional CFTR functional readouts within the lung are necessary.

Transepithelial potential difference measurement is a technique that creates a local voltage measurement circuit that explores the response to imposed ion/electrical gradients that can be modulated with specific channel inhibition or stimulation. These measurements have been made via bronchoscopy in gene therapy trials and have also been used in the nares for early modulator trials or diagnostic purposes. While nasal potential difference (NPD) is not a direct readout of CFTR function in conducting airway epithelial cells, inhaled gene therapy has the potential to transfect the inferior turbinate of the nares and thus may be a non-invasive site for evaluation of *CFTR* delivery (Alton et al., 2016; Sermet-Gaudelus et al., 2021).

While not functional, CFTR expression can be evaluated by brushing the airway via bronchoscopy following gene treatment. CFTR protein including post translational modification is then assessed by western blot. Other sensitive techniques to distinguish between patient *CFTR* alleles and *CFTR* delivered by a gene therapy can include polymerase chain reaction with primers customized to regions affected by an individual’s *CFTR* sequence or liquid chromatography mass spectrometry (LCMS) to measure protein peptides belonging to a specific CFTR variant (Wang H et al., 2022). Finally, expression analyses that identify which cell types are transduced including immunohistochemistry (IHC) along with cell type markers in bronchial tissues are critical to establishing which CFTR functions will be restored (see Figure 3), although antibodies used to detect CFTR by IHC have been wrought with challenges (Sato et al., 2021).

### 6.2 Lung structure

Lung structural changes are predictive of changes in lung function although the two are not interchangeable. High resolution computed tomography (HRCT) chest scanning is the gold standard for detecting lung structural abnormalities and diagnosing bronchiectasis; however, its use of radiation prevents it from being used at more than 1–2 years intervals. Additionally, CT scanning procedures and breathing maneuvers can be variable at different sites making multicenter comparisons challenging. Sedation can be required for young children and previous binary scoring made early lung disease difficult to detect. The recently developed Perth-Rotterdam Annotated Grid Morphometric Analysis for CF (PRAGMA-CF) is a scoring system predicted to be more sensitive to disease progression in the first 3 years of life (Rosenow et al., 2015). PRAGMA-CF CT scores report on percent disease, bronchiectasis, trapped air, mucus plugging and bronchial wall thickening. In the RECOVER study, this method was applied to spirometry-controlled chest CT scans and compared to measurements of ppFEV in people aged 12 and older on ETI modulators. PRAGMA-CF scores reported significant improvements in all aspects except bronchiectasis and ppFEV1 measurements were significantly improved in people on ETI modulators; however, outcomes were not entirely correlated with each other, across genotypes (ΔF508/ΔF508 vs ΔF508/minimal function) or between follow up timepoints (6 vs. 12 months) (McNally et al., 2023).

Recent developments in ultra low dose radiography protocol can evaluate CF lung disease at an equivalent dose to a chest x-ray (Moloney et al., 2021). Furthermore, an additional chest imaging procedure that does not use radiation at all is magnetic resonance imaging (MRI) which can be performed in infants and preschool children under sedation. Global MRI scores which report on morphology, perfusion, airway wall thickening/bronchiectasis, and mucus increased during the first 4 years after diagnosis and were correlated with the rate of pulmonary exacerbations during that time (Stahl et al., 2021).

### 6.3 Lung function

The primary efficacy endpoint for CF clinical trials is demonstrated improvement from baseline in percent predicted forced expiratory volume in one second (ppFEV1) or how much air a person can exhale in one second after deep inhalation. This lung function measurement is reflective of structural changes that occur during development of lung disease. The CFTR modulators have given us a better understanding of achievable outcomes with CFTR therapeutics. The earliest CFTR potentiator Ivacaftor, approved for Class III gating variants demonstrated a robust increase in lung function of 9–10 ppFEV1 over baseline (McKone et al., 2014). The subsequent potentiator/corrector combination, Orkambi that needed to rescue trafficking of misfolded protein and open probability at the cell surface (Class II), showed a significant, though less impressive improvement of 2.6%–4%, when 5% was thought to be meaningful clinical improvement (Wainwright et al., 2015). However, it was also demonstrated that pulmonary exacerbations which require hospitalization were 30%–39% lower with treatment regardless of immediate improvements in lung function. Acute exacerbation events are thought to drive progression of CF lung disease therefore this represents significant improvement in lung health and long-term function.

CF therapies are first tested in adults aged 18 years or older and are expanded to younger people only after safety and efficacy have been demonstrated in adults. This presents a challenge and a high bar for efficacy in a progressive disease where extensive lung remodeling has occurred through recurrent, chronic infections. It raises the question of whether advanced lung disease can be reversed. After approval of Trikafta, the most effective CFTR modulator for minimal function variants, a real-world observational study of ∼500 participants demonstrated that people with <65% predicted FEV1 had a mean improvement of 12.2 ppFEV1 at 6 months, while those with >90% had an improvement of 6.5 ppFEV1 (Nichols et al., 2021a). In a study of people with advanced CF lung disease (<35% ppFEV1), rapid improvement in lung function resulting in +15 ppFEV1 after 1–3 months was exhibited (pooled data), leading to 73% of people wait-listed for lung transplantation being removed from candidate list and 97% of people undergoing evaluation for lung transplantation were no longer eligible due to decreased clinical severity (Burgel et al., 2021). These data demonstrate advanced lung disease can be modified by CFTR functional restoration and that lower levels of FEV1 may still represent meaningful lung improvements.

People younger than age six cannot perform forced spirometry from which ppFEV1 measurements are derived. Therefore, additional measurements of lung function have been explored in adults and children and correlated with FEV1 so they can be used to detect more sensitive changes in younger PwCF. Lung clearance index (LCI) measured by multiple breath washout (MBW) of an inert marker gas is more sensitive than spirometry to early stage lung disease and can be measured repeatedly in children as young as 3.5 years of age (success rate of >80%; (Downing et al., 2016)). LCI was measured in people aged 5–19 years and abnormal readings were detected in 85% of people with evidence of bronchiectasis, a key factor in CF disease, and in 93% of people with an abnormal HRCT. In comparison, FEV1 was reduced in 19% and 26% of PwCF. Alternatively, FEV1 reduction was more specific for lung abnormalities than LCI which was found to be abnormal in one third of poeple with normal HRCT. It’s hypothesized that HRCT may miss distal lung abnormalities identified by LCI (Gustafsson et al., 2008). In the RECOVERY study, LCI was measured in addition to FEV1. While LCI improved at similar rates in those above and below 90 ppFEV1, ppFEV1 improved more dramatically in people that started below 90. These data are concurrent with the hypothesis that LCI can detect lung disease more sensitively in those with preserved lung function (McNally et al., 2023). Now that Trikafta is available for ages 6 years and older a similar study can be repeated in younger children to determine whether treads in LCI and PRAGMA-CF scores are more reliable methods of measuring lung disease in young people.

### 6.4 Lung disease

Additional symptoms of CF lung disease include chronic cough, a result of mucus plugging and bronchiectasis, and cough counting is a measurement that can be used to assess treatment. Self-reported cough counting is burdensome and inaccurate and more accurate measurements can only be derived in a health center. More reliable automated methods of cough counting may improve the usefulness of this measurement in the future (Crooks et al., 2017; Zhang et al., 2022). The chronic infections that plague PwCF, with *Pseudomonas aeruginosa* being the primary pathogen, are also a core measurement in changes in disease status. Bronchial cultures from expectorated sputum or bronchoalveolar lavage fluid are used to measure infection as well as inflammatory cytokines in systemic circulation. Young children with CF do not spontaneously expectorate sputum, even with cough and sputum induction is also being tested to sample the lower airways (Ronchetti et al., 2018). It is interesting to consider than in later stage disease, a reduction in expectorated sputum may also be a readout of lung disease improvement. Finally, the Cystic Fibrosis Questionnaire-Revised (CFQ-R) an important patient reported quality of life measurement, with a section focused on lung health, that is also used in most trials. Figure 5 summarizes potential endpoints in CF trials and how early they can be performed in patients.

## 7 Outlook and conclusion

*CFTR* gene therapy has been on the horizon since the discovery of the *CFTR* gene in 1989. Although many viral and non-viral delivery vectors have been tested, none have yet achieved the transduction, expression, and durability necessary to improve lung function in PwCF. A goal of 10%–25% rescue of CFTR expression by a gene therapy has been derived based on experiments in highly relevant airway *in vitro* models and CF clinical phenotypes of *CFTR* variants that have been sequenced and analyzed for expression and function. High transduction and durable functional restoration are most likely to be achieved by a highly lung tropic vector that can express wild type *CFTR*, and has the potential to either transduce basal stem cell progenitors or to be readministered. Furthermore, a large repertoire of exploratory endpoints testing more sensitive lung function measurements should be included in clinical trials given the complex tasks of CFTR function and how they lead to lung function decline and lung disease. Finally, earlier intervention has the potential to stave off lung inflammation and remodeling that initiate CF lung disease but may also have a positive improvement in durability of the gene therapy without the increase in cellular turnover that drives remodeling. Despite the challenges of developing a *CFTR* gene therapy, advances in delivery vectors, expression enhancers and clinical endpoints provide hope that success is on the horizon.
